# New Strigolactone Mimics as Exogenous Signals for Rhizosphere Organisms

**DOI:** 10.3390/molecules22060961

**Published:** 2017-06-09

**Authors:** Florin Oancea, Emilian Georgescu, Radoslava Matusova, Florentina Georgescu, Alina Nicolescu, Iuliana Raut, Maria-Luiza Jecu, Marius-Constantin Vladulescu, Lucian Vladulescu, Calin Deleanu

**Affiliations:** 1 National Research & Development Institute for Chemistry & Petrochemistry–ICECHIM, Spl. Independentei 202, RO-060021 Bucharest, Romania; Florin.Oancea@icechim.ro (F.O.); iulia_rt@yahoo.com (I.R.); Jecu.Luiza@icechim.ro (M.-L.J.); 2 Research Center Oltchim, St. Uzinei 1, RO-240050 Ramnicu Valcea, Romania; g_emilian@yahoo.com; 3 Plant Science and Biodiversity Center SAS, Institute of Plant Genetics and Biotechnology, PO Box 39A, 95007 Nitra, Slovakia; Radka.Matusova@savba.sk; 4 Research Department Teso Spec S. R. L., Str. Muncii 53, RO-915200 Fundulea, Romania; florentina_fg@yahoo.com (F.G.); marius.vladulescu@tesospec.com (M.-C.V.); lucian.vladulescu@tesospec.com (L.V.); 5 Centre of Organic Chemistry, Romanian Academy, Spl. Independentei 202B, RO-060023 Bucharest, Romania; alina220382@yahoo.com; 6 Institute of Macromolecular Chemistry, Romanian Academy, Aleea Grigore Ghica Voda 41-A, 700487 Iasi, Romania

**Keywords:** strigolactones, strigolactone mimics, natural-products like molecules, exogenous signals

## Abstract

The importance of strigolactones in plant biology prompted us to synthesize simplified strigolactone mimics effective as exogenous signals for rhizosphere organisms. New strigolactone mimics easily derived from simple and available starting materials in significant amounts were prepared and fully characterized. These compounds contain an aromatic or heterocyclic ring, usually present in various bioactive molecules, connected by an ether link to a furan-2-one moiety. The new synthesized strigolactone mimics were confirmed to be active on plant pathogenic fungi and parasitic weed seeds.

## 1. Introduction

More than 25 natural strigolactones (SLs) have been detected and isolated from root exudates of mono- and dicotyledonous plants. SLs are apocarotenoids [1,2,3] functioning as both plant hormones [4,5], endogenous signals which regulate plant response to stress [6,7], and ecomones/semiochemicals, exogenous signals involved into exchange of information in the plant rhizosphere [8,9,10]. Strigol, the first member of the SLs family, was isolated from cotton root exudates and identified as a germination stimulant of the seed of the parasitic plant *Striga lutea* [11]. The function of germination stimulants of parasitic weed seeds in the rhizosphere is only the first of SLs’ recognized functions [12,13,14]. SLs have been also identified as signaling molecules involved in plants’ symbiosis with mycorrhizae (AM) fungi [15,16,17] and in the symbiosis between rhizobia and leguminous plants [18,19,20,21]. Conflicting data regarding the influence of SL analogues on fungal pathogen growth patterns were reported [22,23,24,25,26]. Reactive oxygen species (ROS) and mitochondria have been recently reported as emerging mediators of SLs’ actions on plant pathogenic fungi [23]. SLs were recognized as a new class of plant hormones [4,5,27,28,29,30,31], which regulate plants’ architecture [32,33,34], being also related to plants’ response to stress [6,7,35].

**Figure 1:**
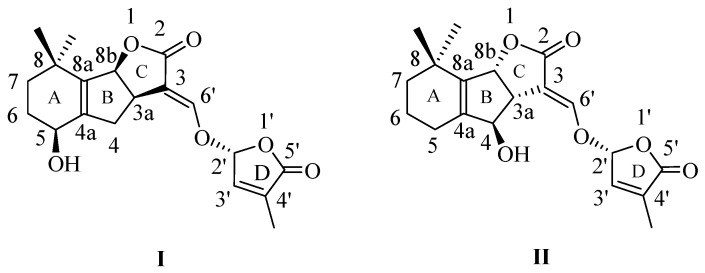
Naturally occurring SL structures.

Naturally occurring SLs have a tricyclic lactone skeleton (ABC rings), attached by an enol ether bond to an α,β-unsaturated furanone part (the D-ring). There are two naturally occurring SL families depending on the BCD part stereochemistry: one in which the stereochemistry is the same as in (+)-strigol (**I**) and the other one having the stereochemistry found in natural (−)-orobanchol (**II**) [36,37] (Figure 1).

The structure–activity relationship of SLs as exogenous signals that stimulate the germination of parasitic plant seeds, revealed that the bioactivity is located in the CD part of the SL molecule [37,38,39], and the presence of the methyl group at C-4’ of the D-ring and the presence of an enol ether combined with a carbonyl group of a ketone or of an ester, linked to the D-ring are essential for their bioactivity [37]. The substitution of the ether oxygen by nitrogen or sulfur atom does not appear to affect the bioactivity.

**Figure 2:**
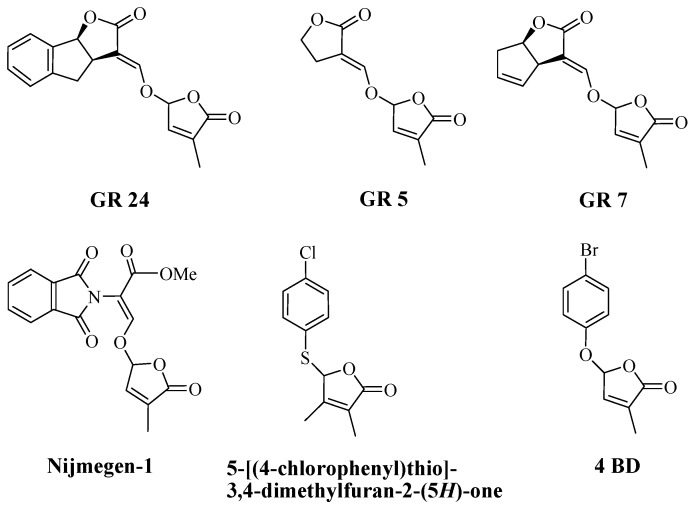
Chemical structures of several synthetic SL analogues and mimics.

Natural SLs can be obtained in minute amounts from plant root exudates or by long multistep chemical syntheses. Simplified synthetic SL analogues and mimics were developed as natural product-like small molecules, in order to investigate the structure–activity relationship for their various activities in plants. SL analogues have a simplified structure that retained the bioactophore CD part of natural SLs [40,41,42,43,44]. Among these, GR24 (Figure 2), prepared firstly by Johnson et al. [43], is considered as the standard for SL analogues and its synthetic procedure was improved and further developed later by other groups [44].

SL mimics are an important group of simple compounds that mimic the activity of SLs. SL mimics do not have the typical SL structural requirements of ABC rings attached by an enol ether bond to a D-ring. Usually, SL mimics have only an aryloxy-, arylthia- or aroyloxy-substituent at C-5 of the D-ring (Figure 2) [30,31,37,45,46,47,48,49,50]. The mode of action was explained by the interaction of these SL mimics with a protein receptor which led probably to the enzymatic hydrolytic elimination of the D-ring as HO-D. This first step generates a sequence of reactions at the receptor site and triggers the signal transmission [50,51,52,53]. However, the protein receptors of AM fungi have not been yet isolated and identified.

SLs represent an important research field which may lead to agricultural innovation [35,49,54]. The SLs importance in plant biology, both as exo- and endo-signals, prompted us to synthesize simplified SL mimics easily accessible in significant amounts. Our group is interested in the synthesis of new potentially bioactive compounds [55,56,57] and as part of our ongoing research related to such molecules, we report here the synthesis of some new SL mimics, derived from simple and available starting materials, containing an aromatic or heteroaromatic nucleus connected by an ether link to a furan-2-one moiety. We also report their biological activity as exogenous signals for rhizosphere organisms, parasitic weed seeds and plant pathogenic fungi.

## 2. Results and Discussion

### 2.1. Synthesis of New Strigolactone Mimics

Our new SL mimics, derived from simple and available starting materials, contain an aromatic or heteroaromatic ring, present in various bioactive molecules, linked by an ether group to a furan-2-one moiety. The common features of new synthesized compounds are a bioactive (hetero)aromatic core and the presence of a D ring that can increase the pre-existing activity.

**Scheme 1:**
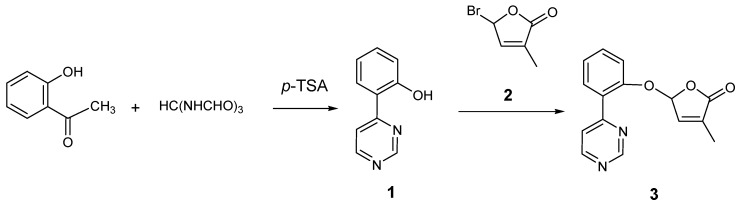
Synthesis of SL mimic **3** derived from 4-(2-hydroxyphenyl)pyrimidine (**1**).

The SL mimic 3-methyl-5-(2-pyrimidin-4-yl-phenoxy)-5*H*-furan-2-one (**3**) was obtained by the coupling reaction of 4-(2-hydroxyphenyl)pyrimidine (**1**) with 5-bromo-3-methyl-5*H*-furan-2-one (**2**) in basic medium. The starting 4-(2-hydroxyphenyl)pyrimidine (**1**) was prepared for the first time, according to a previously reported method [58], by treating 2-hydroxyacetophenone with trisformylaminomethane (Scheme 1).

**Scheme 2:**
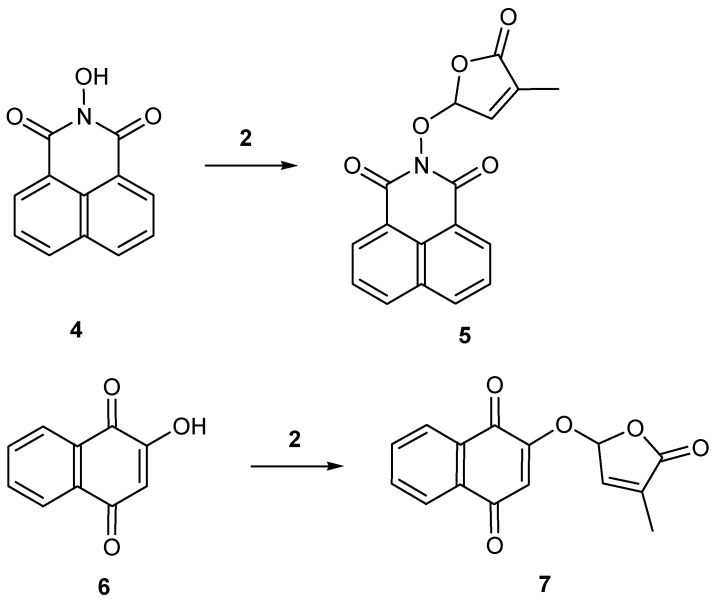
Synthesis of synthetic SLs derived from cyclic hydroxy ketones.

The other two synthetic SLs were obtained by treating commercially available cyclic hydroxyl-ketones in DMF with 5-bromo-3-methyl-5*H*-furan-2-one (**2**) in the presence of K_2_CO_3_ (Scheme 2). Thus, the coupling reaction of 2-hydroxy-benzo[de]isoquinoline-1,3-dione (**4**) with 5-bromo-3-methyl-5*H*-furan-2-one (**2**) led to the SL mimic 3-methyl-5-(benzo[de]isoquinoline-1,3-dione-2-yloxy)-5*H*-furan-2-one (**5**), bearing a naphthalimide core linked by an ether group to the furan-2-one ring. Numerous compounds containing a *N*-substituted phthalimide nucleus are known as plant growth regulating chemicals [59,60,61], as well as compounds regulating the activity of gibberellins A4 and A7 [62,63]. In the same way, by treating a cyclic hydroxyketone, namely 2-hydroxy-1,4-naphthoquinone (**6**), with 5-bromo-3-methyl-5*H*-furan-2-one (**2**) the SL mimic 3-methyl-5-(1,4-naphthoquinone-2-yloxy)-5*H*-furan-2-one (**7**) was obtained.

The newly synthesized compounds were structurally characterized by their chemical and spectral data. The synthesized compounds have only one asymmetric carbon atom, thus there are no particular stereochemical issues to be solved. All compounds produced in the above described reactions were obtained as racemates.

The progress of the coupling reactions may be followed in the ^1^H-NMR spectra of the synthesized compounds by the disappearance of the characteristic OH group signal from 10–13 ppm and the appearance of the three signals corresponding to the 5-bromo-3-methyl-5*H*-furan-2-one moiety. The detailed NMR data (chemical shifts and coupling constants) for each of the synthesized compounds are presented in the Materials and Methods Section, while the NMR spectra are presented as Supplementary Materials.

### 2.2. The Biological Activity of Synthesized Compounds

#### 2.2.1. The Effect on Parasitic Weed Seeds

**Figure 3:**
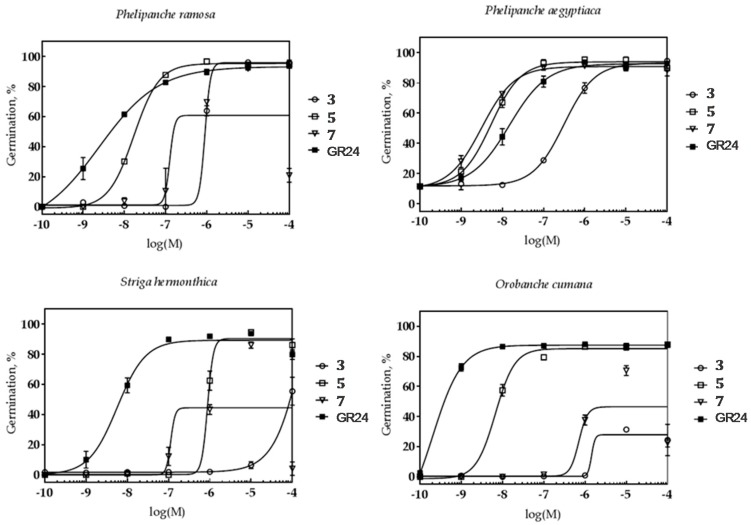
Effect of new synthetic compounds **3**, **5**, **7** and reference synthetic analogue GR24 on germination rate of *Phelipanche ramosa*, *P. aegyptiaca*, *Orobanche cumana* and *Striga hermonthica*. Germination assays were performed in triplicate, over a range of SL concentrations. Data are the mean ± SE. Logistic dose-response curves were gained by nonlinear regression, with variable Hill coefficient.

The effect of the synthesized SLs on the induction of parasitic weed seed germination was tested. The germination bioassays were carried out according to the previously described method [64] on *Phelipanche ramosa*, *Phelipanche aegyptiaca*, *Orobanche cumana* and *Striga hermonthica* seeds. The results clearly demonstrated that compounds **3**, **5**, **7** and reference synthetic SL analogue GR24 (racemic mixture), induced germination of the seeds. Results also showed the different activity of the compounds on different parasitic weed seeds (Figure 3). The applied doses covered a broad range of responses from no germination to toxic effects in the case of analogue **7** (10^−4^ M) for *S. hermonthica*.

Low, but significant (11%) spontaneous germination for *P. aegyptiaca* seeds was observed (demineralized water, or demineralized water plus 3% acetone as negative controls). Spontaneous germination was rarely observed for *P*. *ramosa*, *O. cumana* and *S. hermonthica*. The synthetic mimic **3** induced germination at concentrations ranging from 10^−8^ M for *P. aegyptiaca* to 10^−5^ M for *S. hermonthica*. Compound **3** induced 55% germination of *S. hermonthica* seeds, but with relatively high dose of compound (10^−4^ M), in comparison to the dose which is necessary to induce 50% of maximum germination [GS]_50_ by the reference analogue GR24 (5.7 × 10^−9^ M). The lowest germination activity of **3** was observed for *O. cumana* seeds, with maximal germination induced (*R_max_* = 28%) and [GS]_50_ 1.44 × 10^−6^ M. The compound **3** was more potent for *P. ramosa* with *R_max_* = 96% and [GS]_50_ 9 × 10^−7^ M and *P. aegyptiaca* with *R_max_* = 93% and [GS]_50_ 3 × 10^−7^ M. The synthetic mimic **5** was an active germination stimulant for broomrapes, with [GS]_50_ 1.7 × 10^−8^ M for *P. ramosa*, 5.3 × 10^−9^ M for *P. aegyptiaca* and 6.7 × 10^−9^ M for *O. cumana.*

Compound **5** induced maximal germination in these species, comparable to the maximal germination ability of GR24. For *S. hermonthica*, a higher concentration of compound (10^−6^ M) was needed to reach maximal seed germination. Synthetic compound **7** was the least potent among all analogues tested at inducing germination of *P. ramosa*, *S. hermonthica* and *O. cumana*, with maximal germination induced by **7** reaching 61%, 47% and 28% for these species. At high dose (10^−4^ M) **7** greatly reduced germination of *P. ramosa* seeds (21%) and caused no germination of *S. hermonthica* seeds. Our results indicate different affinity of the compounds to the putative receptor(s) which is expressed by [GS]_50_ values. In general, **3** exhibits the lowest affinity to the receptor(s)—about 10 to 100,000 times less active then GR24 in the species studied—while **5** is the most potent compound for induction of parasitic weed seed germination out of new synthetic SL mimics tested.

#### 2.2.2. The Effect on Plant Pathogenic Fungi

**Figure 4:**
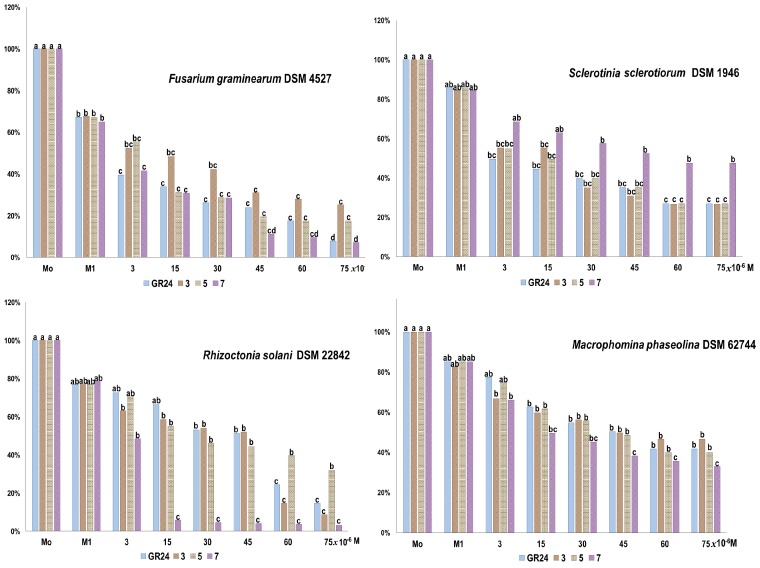
Effect of new synthetic SL mimics **3**, **5**, **7** and reference synthetic analogue GR24 on radial growth of water–agar 1.8% of *F. graminearum* (DSM 4527), *S. sclerotiorum* (DSM 1946), *R. solanii* (DSM 22842) and *M. phaseolina* (DSM 62744). Least-significant differences (LSD) were calculated and means were compared. The different letters indicate significant differences (*p* < 0.05). M_0_—water-agar 1.8%; M_1_—water agar containing 0.5% (*w*/*v*) acetone.

The synthetic SL mimics inhibited the growth of the tested fungal plant pathogens, the effect being visible from the lowest concentration (3 × 10^−6^ M, Figure 4). Generally, compounds **3** and **5** display a similar response as GR24 on *S. sclerotiorum* (DSM 1946) and *M. phaseolina* (DSM 62744) as the responses of these tested fungi to compounds **3** and **5** are on the same level with those reported already for the strains of the same genera treated with GR24 [22]. Compound **3** is less effective than other compounds on *F. graminearum* (DSM 4527). However, it is the most active compound on *R. solanii* (DSM 22842). Compound **5** has an almost similar response to GR24 on *F. graminearum* (DSM 4527) inhibition and is the least active compound on *R. solanii* (DSM 22842). Compound **7** is very active on *R. solanii* (DSM 22842), being more active than the reference compound GR24. On *M. phaseolina* (DSM 62744) compound **7** is the most active compound at concentrations higher than 15 × 10^−6^ M and on *F. graminearum* (DSM 4527) is displays a response like GR24, however, it is the least active from the tested SL mimics on *S. sclerotiorum* (DSM 1946).

There are conflicting data in the literature regarding the influence of synthetic SL mimics on fungal plant pathogen development patterns. Thus, GR24 was reported to inhibit radial fungal growth when embedded into agar media [22,23]. However, GR24 spread from an acetone solution on the surface of the agar media [24], or which is diffusing from small holes in agar medium [25] or from small fiberglass discs deposited on the surface of the agar media [26] were reported to have no effects on the growth of fungal plant pathogens.

Experimental conditions thus clearly influence the results, and availability of the rather hydrophobic SL chemical analogues on rather hydrophilic (agar) medium being essential for better understanding the interactions between synthetic SLs and/or the naturally occurring SLs and plant fungal pathogens. A better understanding of SLs bioavailability on hydrophilic/amphiphilic media could support also further optimization of SLs application as soil treatment. The tested compounds produced an increase of branching activities (Table 1).

The tested SL synthetic mimics, at the highest concentration (75 × 10^−6^ M) determine the most significant increase in hyphal branching on *S*. *sclerotiorum* (DSM 1946). This is similar to the result reported for another *S. sclerotiorum* strain as response to GR24 [22]. A lower hyphal branching was determined by the tested synthetic SL compounds on *R. solanii* (DSM 22842) and *F. graminearum* (DSM 4527). However, *M. phaseolina* (DSM 62744) has an opposite response to the tested synthetic SL mimics, the hyphal branching being depressed by all tested compounds. The responses of tested fungal strains to compound **5** are quite similar to the response to GR24. Compound **7** determines a less significant hyphal branching response and compound **3** determines an intermediate response.

The effects on plant pathogenic fungi were reported till now only for SL analogues (i.e., GR24). We report here the first evidence of the effects for SL mimics on plant pathogenic fungi. This suggests the existence on plant pathogenic fungi of a SL perception system able to realize the enzymatic hydrolytic elimination of the D-ring as HO-D.

### 2.3. Chemical Stability

**Figure 5:**
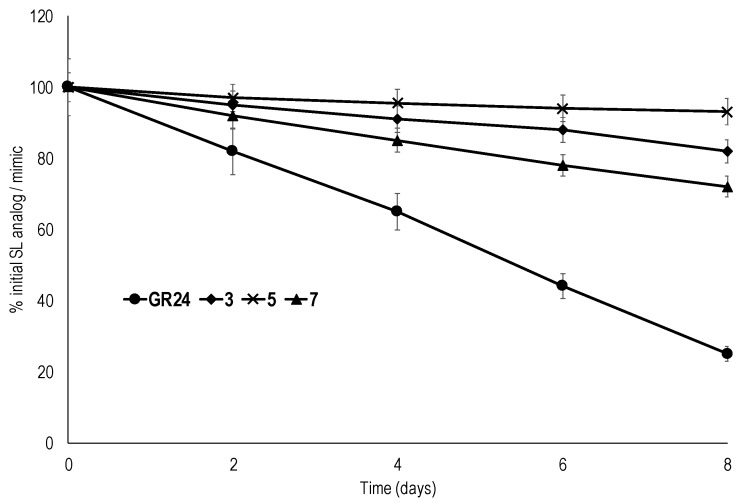
Chemical stability of SL mimics, (+/−)**3**, (+/−)**5**, (+/−)**7** and (+/−)GR24 at 2 °C, pH 6.7 on aqueous solutions of 50 µg/mL, done by dilution of acetone stock solution into an ethanol:water mixture (7:30, *v*/*v*). Data are represented as means ±SE (*n =* 3).

The stability of our SL mimics was verified in a known test in aqueous solution, acetone-ethanol-water [30]. This aqueous solution test demonstrated the following order **5** > **3** > **7** > GR24 (Figure 5), our SL mimics showing a good stability.

Synthetic SLs with good stability in aqueous solution, compared to GR24, were already reported [30,31]. However, these synthetic SLs, reported as stable in aqueous solution, have a low activity as exo-signals in the rhizosphere [31]. Our SL analogues and mimics, stable in aqueous solution, on the other hand, have a significant activity as exogenous signals for rhizosphere organisms.

## 3. Materials and Methods

### 3.1. General Information

The melting points have been determined on a Boetius apparatus (Carl Zeiss, Jena, Germany) and are uncorrected. A Nicolet Impact 410 spectrometer (Thermo Scientific, Waltham, MA, USA) was used for recording the IR spectra in KBr pellets. The NMR analysis was performed on an Advance III 400 spectrometer (Bruker BioSpin, Rheinstetten, Germany), operating at 400.1, 100.6 and 40.6 MHz for ^1^H, ^13^C and ^15^N, respectively. The 1D and 2D spectra were recorded with a 5 mm multinuclear inverse detection z-gradient probe. Unambiguous 1D NMR signal assignments were made based on 2D NMR homo—and heteronuclear correlations. H-H COSY, H-C HSQC and H-C HMBC spectra were recorded using standard pulse sequences in the version with z-gradients, as delivered by Bruker with TopSpin 2.1 PL6 spectrometer control and processing software. The ^15^N chemical shifts were obtained as projections from the 2D indirectly detected H-N HMBC spectra, employing a standard pulse sequence in the version with z-gradients as delivered by Bruker (TopSpin 2.1 PL6). Chemical shifts are reported in δ units (ppm) and were referenced to the residual solvent signals (for CDCl_3_^1^H at 7.26 ppm and ^13^C at 77.0 ppm and for DMSO*-d*_6_^1^H at 2.51 ppm and ^13^C at 39.4 ppm). The ^15^N chemical shifts are referenced to liquid ammonia (0.0 ppm) using nitromethane (380.2 ppm) as external standard. Satisfactory microanalyses for all new compounds were obtained: C ± 0.20, H ± 0.16, N ± 0.26. All other reagents, aside from the starting material **1** which preparation is given below, and the common intermediate 5-bromo-3-methyl-5*H*-furan-2-one (**2**), which was obtained in good yield (81%) by the bromination of 3-methyl-5*H*-furan-2-one with *N*-bromosuccinimide in CCl_4_ [65] and was used as crude reaction product, were commercial products (Sigma-Aldrich, Steinheim, Germany) used without any additional purification.

### 3.2. Preparation of 4-(2-Hydroxyphenyl)pyrimidine (***1***)

Starting material **1** was prepared by treating 2-hydroxyacetophenone with trisformyl-aminomethane according to a previously reported method for the synthesis of 4-phenylpyrimidine [58]. Pale yellow solid, mp 116–118 °C. Yield 61%. Anal. Calcd. for C_10_H_8_N_2_O (172.18): C, 69.75; H, 4.68; N, 16.27%. Found: C, 69.92; H, 4.75; N, 16.20%. FT-IR (KBr): 3053, 1581, 1538, 1504, 1480, 1417, 1284, 1240, 1157 cm^−1^. ^1^H-NMR (DMSO-*d*_6_), δ (ppm): 6.96–7.01 (m, 2H, H-3’ and H-5’), 7.43 (td, *J* = 6.9 Hz, 1.6 Hz, 1H, H-4’), 8.11 (dd, *J* = 7.8 Hz, 1.4 Hz, 1H, H-6’), 8.26 (dd, *J* = 5.6 Hz, 1.3 Hz, 1H, H-6), 8.90 (d, *J* = 5.6 Hz, 1H, H-5), 9.25 (d, *J* = 1.1 Hz, 1H, H-2), 12.80 (s, 1H, OH). ^13^C-NMR (DMSO-*d*_6_), δ (ppm): 117.3 (CH-6), 117.9 (CH-3’), 118.0 (C-1’), 119.4 (CH-5’), 128.4 (CH-6’), 133.2 (CH-4’), 156.7 (CH-2), 158.0 (CH-5), 159.3 (C-2’), 162.9 (C-4). ^15^N-NMR (DMSO-*d*_6_), δ (ppm): 268.1 (N-3), 289.5 (N-1).

### 3.3. Typical Procedure for the synthesis of SL mimics

To a solution of a hydroxy derivative **1**, **4** or **6** (10 mmol) in DMF (15 mL) was added K_2_CO_3_ (1.52 g, 11 mmol), followed by the slow addition under stirring at room temperature of the crude 5-bromo-3-methyl-5*H*-furan-2-one (**2**, 2.21 g, 12.5 mmol). The resulting mixture was continuously agitated at room temperature for the next 24 h. The reaction mixture was then poured into water (80 mL) and extracted with CH_2_Cl_2_ (3 × 75 mL). The combined extracts were washed with an equal volume of water and dried over anh.Na_2_SO_4_. The solvent was partly removed under reduced pressure and the solid formed was filtered and recrystallized to obtain SL compounds **3** and **7**, respectively. For the separation of compound **5** the solvent was removed under reduced pressure, the residue was chromatographed on a SiO_2_-packed column eluting with EtOAc:hexane 25% and finally recrystallized.

### 3.4. Product Characterization

*3-Methyl-5-(2-pyrimidin-4-yl-phenoxy)-5H-furan-2-one* (**3**). Pale yellow crystals, m.p. 215–217 °C (from CHCl_3_/MeOH). Yield 39% (1.05 g). Anal. Calcd. for C_15_H_12_N_2_O_3_ (268.27): C, 67.16; H, 4.51; N, 10.44%. Found: C, 67.29; H, 4.55; N, 10.52%. IR (KBr): 1765, 1726, 1575, 1538, 1477, 1416, 1388, 1242, 1209, 1097, 1017 cm^−1^. ^1^H-NMR (CDCl_3_), δ (ppm): 1.97 (t, *J* = 1.4 Hz, 3H, CH_3_), 6.30 (quintet, *J* = 1.2 Hz, 1H, H-5), 6.89 (quintet, *J* = 1.5 Hz, 1H, H-4), 7.21 (td, *J* = 7.3 Hz, 1.0 Hz, 1H, H-5’), 7.38 (dd, *J* = 8.1 Hz, 0.5 Hz, 1H, H-3’), 7.47 (td, *J* = 7.4 Hz, 1.7 Hz, 1H, H-4’), 7.73 (dd, *J* = 5.3 Hz, 1.4 Hz, 1H, H-6”), 7.86 (td, *J* = 7.7 Hz, 1.6 Hz, 1H, H-6’), 8.70 (d, *J* = 5.3 Hz, 1H, H-5”), 9.25 (d, *J* = 1.2 Hz, 1H, H-2”). ^13^C-NMR (CDCl_3_), δ (ppm): 10.6 (CH_3_), 99.4 (CH-5), 116.6 (CH-3’), 121.8 (CH-6”), 124.2 (CH-5’), 127.6 (C-1’), 131.1 (CH-6’), 131.9 (CH-4’), 134.7 (C-3), 141.9 (CH-4), 154.4 (C-2’), 156.4 (CH-5”), 158.6 (CH-2”), 162.5 (C-4”), 170.9 (CO). ^15^N-NMR (CDCl_3_), δ (ppm): 281.4 (N-3”), 290.6 (N-1”).

*3-Methyl-5-(benzo[de]isoquinoline-1,3-dione-2-yloxy)-5H-furan-2-one* (**5**). Beige solid, yield 78%, 2.41 g, m.p. 236–238 °C (from acetone + MeOH). Anal. Calcd. for C_17_H_11_NO_5_ (309.28): C, 66.02; H, 3.58; N, 4.53%. Found: C, 66.16; H, 3.51; N, 4.47%. IR (KBr, ν_max_): cm^−1^ 1789, 1726, 1688, 1583, 1355, 1341, 1231, 1188, 1097, 1016.^1^H-NMR (CDCl_3_), δ (ppm): 2.02 (s, 3H, CH_3_), 6.48 (s, 1H, H-5), 7.18 (s, 1H, H-4), 7.79 (t, *J* = 7.8 Hz, 2H, H-6’), 8.26 (d, *J* = 8.1 Hz, 2H, H-7’), 8.64 (d, *J* = 7.3 Hz, 2H, H-5’). ^13^C-NMR (CDCl_3_), δ (ppm): 10.8 (CH_3_), 103.2 (CH-5), 122.5 (2 × C-4’), 127.1 (2 × CH-6’), 127.5 (C-9’), 131.8 (C-8’), 132.1 (2 × CH-5’), 134.9 (2 × CH-7’), 136.0 (C-3), 139.9 (CH-4), 160.8 (2 × CO-3’), 170.7 (CO-2).

*3-Methyl-5-(1,4-naphthoquinone-2-yloxy)-5H-furan-2-one* (**7**). Beige solid, yield 51%, 1.38 g, m.p. 153–155 °C (from CHCl_3_/MeOH). Anal. Calcd. for C_15_H_10_O_5_ (270.24): C, 66.67; H, 3.73%. Found: C, 66.75; H, 3.78%. IR (KBr, ν_max_): cm^−1^ 3096, 3049, 1785, 1679, 1657, 1614, 1593, 1349, 1331, 1303, 1266, 1244, 1205, 1091, 1017. ^1^H-NMR (CDCl_3_), δ (ppm): 2.03 (s, 3H, CH_3_), 6.35 (s, 1H, H-5), 6.63 (s, 1H, H-3’), 7.12 (s, 1H, H-4), 7.73 (t, *J* = 7.2 Hz, 1H, H-8’), 7.77 (t, *J* = 7.2 Hz, 1H, H-7’), 8.08 (d, *J* = 6.8 Hz, 1H, H-6’), 8.12 (d, *J* = 7.1 Hz, 1H, H-9’). ^13^C-NMR (CDCl_3_), δ (ppm): 10.7 (CH_3_), 96.7 (CH-5), 114.6 (CH-3’), 126.3 (CH-6’), 126.8 (CH-9’), 130.8 (C-10’), 131.7 (C-5’), 133.7 (CH-8’), 134.5 (CH-7’), 135.4 (C-3), 141.2 (CH-4), 156.9 (C-2’), 170.1 (CO-2), 179.3 (CO-1’), 184.4 (CO-4’).

### 3.5. Biological Activity Tests

#### 3.5.1. Germination Bioassay

##### Preparation of Seeds

The biological activity of the new synthetic SL mimics was assayed on *P. ramosa*, *P. aegyptiaca*, *O. cumana* and *S. hermonthica* seeds. The seeds of broomrapes and witchweed require pre-conditioning/warm stratification. Seed pre-conditioning was done under sterile conditions, according to Matusova et al. [64]. Seeds were briefly surface sterilized, by immersion for 5 min in 2% sodium hypochlorite, which contained 0.02% (*v*/*v*) Tween 20, and then they were repeatedly washed with sterile water. Seeds were dried on laminar air flow. Around 100 seeds were distributed on 8 mm (diameter) discs, made from glass fibre filter paper (GFFP). Twelve discs with parasitic weed seeds were placed on previously prepared 9 cm Petri dishes. These prepared Petri dishes were obtained by layering 2 sheets of Whatman filter paper and humidifying them with 3 mL of sterile demineralized water. The Petri dishes with GFFP discs seeds were sealed with Parafilm and incubated. The incubation, necessary to break the dormancy, was done in darkness at 21 °C for 12 days for broomrape seeds and in darkness at 30 °C for 10 days for *S. hermonthica* seeds [64].

##### Preparation of Test Solutions

All compounds were dissolved in acetone to make 3 mM stock solutions and diluted with sterile demineralized water to get appropriate concentrations. All test solutions were prepared just before use.

##### Bioassay

GFFP discs with seeds conditioned, for the above indicated period, were aseptically transferred to new Petri dishes with moistened Whatman filter paper and dried in an air flow cabinet, to remove excess humidity as we previously described [64]. The tested synthetic SL mimics **3**, **5**, **7** and reference analogue GR24 (racemic mixture, Chiralix B.V., Nijmegen, The Netherlands), at concentrations of 100, 10, 1, 0.1, 0.01 and 0.001 µM, were added to three replicate disks, in a volume of 40 µL per disk. In each bioassay, demineralized water and aqueous solution of 3% acetone were used as negative controls. Petri dishes were sealed with Parafilm before incubation. The incubation was done in darkness, at 25 °C for 5 days for broomrape seeds and at 30 °C for 2 days for witchweed seeds. The number of germinated and nongeminated seeds was determined by counting them, after magnification on a DV4 stereomicroscope (Carl Zeiss) A parasitic weed seed was considered as germinated when the radicle was easy to observe, because it clearly protruded the seed coat.

##### Calculation of Logistic Dose–Response Curves.

The calculation of the logistic dose–response curves allows a quantitative description of the sensitivity of the seeds to the tested SL mimics as germination stimulants. This calculation was made with non-linear regression and variable Hill coefficient [64] using GraphPad Prism version 7.02 software (GraphPad Software, La Jolla, CA, USA). Three sensitivity parameters were calculated and used for data analysis: *R_max_*, the maximum seeds germination; [GS]_50_, the dose necessary to induce 50% of maximum seeds germination; *p*, the interaction (Hill) coefficient. The mathematical expression relating the response of seeds to the dose concentrations is:

(1) $$R=R_{min}+\frac{R_{max}-R_{min}}{1+{\left(\frac{{\left[GS\right]}_{50}}{\left[GS\right]}\right)}^{p}}$$

where: *R_min_* is the germination in the absence of the tested germination stimulants (GS); *R_max_* is the maximum germination; [GS] is the dose of the tested SL mimics applied on the experiments; [GS]_50_ is the dose giving 50% response [64].

#### 3.5.2. Test on Plant Pathogenic Fungi

##### Biological Material

The biological activity of the synthetic SL mimics was assayed on the following strains of soil-borne plant pathogens, purchased from DSMZ (The Leibniz Institute DSMZ—German Collection of Microorganisms and Cell Cultures, Braunschweig, Germany): *Fusarium graminearum* Schwabe (DSM 4527); *Sclerotinia sclerotiorum* (Libert) De Bary (DSM 1946); *Macrophomina phaseolina* (Tassi) Goid (DSM 62744) and *Rhizoctonia solani* Kühn (DSM 22842). The fungi were initially cultivated on potato–dextrose agar (PDA) medium (Difco Laboratories, Becton, Dickinson and Company, Franklin Lakes, NJ, USA). The PDA medium was distributed in 90 mm Petri dishes, inoculated with fungi and incubated for 4 days at 25 °C.

##### Preparation of Test Solutions

The stock solution of synthetic SL mimics **3**, **5** and **7** were prepared by dissolving 5 mg in 1 mL acetone. From this stock solution, the working solutions were prepared by dilution with acetone. The resulting synthetic SLs working solutions were further dissolved into sterile, warm and liquid (60 °C) water agar 1.8% (Difco Laboratories), 0.5 mL synthetic SLs solution into 99.5 mL warm and liquid water agar 1.8%. Final concentrations of the SL mimics in agar were: 3, 15, 30, 45, 60, 75 × 10^−6^ M.

##### Bioassay

Influence of the new synthetic SL mimics on growth pattern of plant pathogenic fungi was tested according to Dor et al. [22]. Briefly, 4 mL warm water agar 1.8%, containing new synthetic compounds were spread on a 47 mm Petri dishes (EMD Millipore, Billerica, MA, USA). The Petri dishes were cooled. At the centre of each 47 mm Petri dish, with water-agar and various concentrations of the tested new compounds, it was placed a small piece of mycelium-agar, taken from the edge of each of the tested fungal plant pathogens colony, developed for 4 days at 25 °C on PDA. The inoculated Petri dishes were incubated at 25 °C for 3 days. After 3 days, the developed fungal colonies were observed. The diameters were measured and the resulting areas of the fungal colonies were calculated. The Petri dishes were examined on an inverted microscope (Axiovert 40 CFL, Carl Zeiss). On each primary branch, there were determined the number of hyphal branches, total and branches of different orders, from second till fifth, on a total length of 1000 µm. As negative controls there were used water agar and water agar with 0.5% (*v*/*v*) of the solvent used for SL analogue and mimics solutions (acetone). As positive control reference, synthetic SL analogue GR24 was used, in similar final concentrations of 3, 15, 30, 45, 60, 75 × 10^−6^ M.

##### Statistical Analysis

The obtained data were statistically processed by analysis of variance (JMP^®^ v13.0, SAS Institute Inc., Cary, NC, USA). Least-significant differences (LSD) were calculated and compared with the differences between the means, based on Tukey–Kramer method (*p* < 0.05). Each experiment was repeated three times. Each experiment was done in six replicates. Data presented are the average of the three experiments.

### 3.6. Chemical Stability Tests

The tests for chemical stability of the newly synthesized SL mimics were done in aqueous solutions, with a final concentration of 50 µg/mL. The method described by Boyer et al. [30] was followed. Stock solution (1 mg/mL) of the tested SL analogues and mimics were prepared in acetone. From this stock solution, 20 µL were distributed into HPLC filter vials through 0.2 µm nylon filters (Thomson SINGLE StEP Standard Filter Vials, Restek, Bellefonte, PA, USA), which were further diluted till 400 µL, with 70 µL ethanol, 10 µL indanol solution 1 mg/mL in acetone (indanol being used as internal standard) and 300 µL water. The pH of the resulted SL analogues and mimics aqueous solution was 6.7. The HPLC filter vials were incubated at 21 °C. Each second day, the SL analogue and mimics aqueous solutions from sampled vials were filtered and supernatants were assayed by UPLC analysis, on an Acquity UPLC H-Class System (Waters, Milford, MA, USA) equipped with a Photodiode Array (PDA) Detector, using an Acquity UPLC HSS C_18_ column (1.8 μm, 2.1 × 50 mm). The elution phase for the first 30 s was 5% acetonitrile in water with 0.1% formic acid for 0.5 min. For the next 6 min a gradient from 5% to 100% acetonitrile in water with 0.1% formic acid was applied, followed by 100% acetonitrile with 0.1% formic acid for 3 min. The operating temperature was 40 °C and the flow rate was 0.6 mL/min. The quantification of the non-degraded SL analogues and mimics was done by peaks integration and comparison with the peak of the stable internal standard, indanol. GR24 (racemic mixture, Stichting Chemiefonds Paddepoel, Malden, The Netherlands) was used as reference SL analogue.

## 4. Conclusions

New SL mimics containing an aromatic or heteroaromatic ring, present in various bioactive molecules, connected by an ether link to a furan-2-one moiety were easily synthesized and completely characterized. These affordable compounds were investigated for their activity as active ingredients for plant protection products on plant pathogenic fungi and parasitic weed seeds. The new compounds induced germination of seeds of *Orobanche*, *Phelipanche* and *Striga* spp. and proved biological activities comparable with GR 24 analogue on plant pathogenic fungi.

## Figures and Tables

**Table 1:** The effect of the tested SL synthetic mimics, at 75 × 10^−6^ M concentration, on fungal plant pathogen branching.

Tested Fungi	Water Control	Acetone Control	GR24	3	5	7
*Fusarium graminearum* (DSM 4527)	1.67 (a)	1.33 (a)	5.83 (c)	4.50 (bc)	5.16 (c)	2.83 (ab)
*Sclerotinia sclerotiorum* (DSM 1946)	2.83 (a)	3.33 (a)	20.66 (c)	13.83 (b)	21.33 (c)	12.33 (b)
*Rhizoctonia solanii* (DSM 22842)	4.50 (a)	3.67 (a)	6.83 (ab)	6.67 (ab)	10.67 (b)	7.83 (ab)
*Macrophomina phaseolina* (DSM 62744)	17.83 (a)	12.33 (ab)	3.60 (c)	5.33 (c)	6.67 (bc)	7.33 (bc)

On each primary branch, the number of hyphal branches, total and branches of different orders, from second till fifth, on a total length of 1000 µm were determined. As negative controls, water agar and water agar with 0.5% (*v*/*v*) solvent (acetone) have been used. Least-significant differences (LSD) were calculated and compared with the differences between the means, based on Tukey–Kramer method (*p* < 0.05). Numbers followed by the same letter in brackets do not significantly differ (*p* < 0.05).

## Supplementary Materials

Supplementary materials are available online. The ^1^H- and ^13^C-NMR spectra for compounds **3**, **5**, **7** are available as supplementary material.
