# Supplementary material for: New Strigolactone Mimics as Exogenous Signals for Rhizosphere Organisms
Source: Molecules. 2017 Jun 9;22(6):961. doi: 10.3390/molecules22060961 (PMC6152683; doi:10.3390/molecules22060961)
Supplement: Supplementary file 1 [file molecules-22-00961-s001.pdf]

Supplementary Material

New strigolactone mimics as exogenous signals for rhizosphere organisms

Supporting Information

The ^{1}H NMR and ^{13}C NMR spectra of compounds 3, 5 and 7.

Florin Oancea[1], Emilian Georgescu[2], Radoslava Matusova [3], Florentina Georgescu[4], Alina Nicolescu [5,6], Iuliana Raut [1], Maria-Luiza Jecu[1], Marius-Constantin Vladulescu[4], Lucian Vladulescu[4] and Calin Deleanu[5,6,*]

1 National Research & Development Institute for Chemistry & Petrochemistry – ICECHIM, Spl. Independentei 202, RO-060021 Bucharest, Romania; Florin.Oancea@icechim.ro;iulia_rt@yahoo.com;Jecu.Luiza@icechim.ro
2 Research Center Oltchim, St. Uzinei 1, RO-240050 RamnicuValcea, Romania;g_emilian@yahoo.com
3 Plant Science and Biodiversity Center SAS, Institute of Plant Genetics and Biotechnology, PO Box 39A, 95007 Nitra, Slovakia; Radka.Matusova@savba.sk
4 Research Dept., Teso Spec S. R. L., Str. Muncii 53, RO-915200 Fundulea, Calarasi, Romania; florentina_fg@yahoo.com; marius.vladulescu@tesospec.com; lucian.vladulescu@tesospec.com
5 "C. D. Nenitescu" Centre of Organic Chemistry, Romanian Academy, Spl. Independentei 202B, Bucharest RO-060023, Romania; alina220382@yahoo.com; calin.deleanu@yahoo.com
6 "Petru Poni" Institute of Macromolecular Chemistry, Romanian Academy, Aleea GrigoreGhicaVoda 41-A, 700487 Iasi, Romania;alina220382@yahoo.com; calin.deleanu@yahoo.com
* Correspondence: calin.deleanu@yahoo.com; Tel.: +40-744-340-456

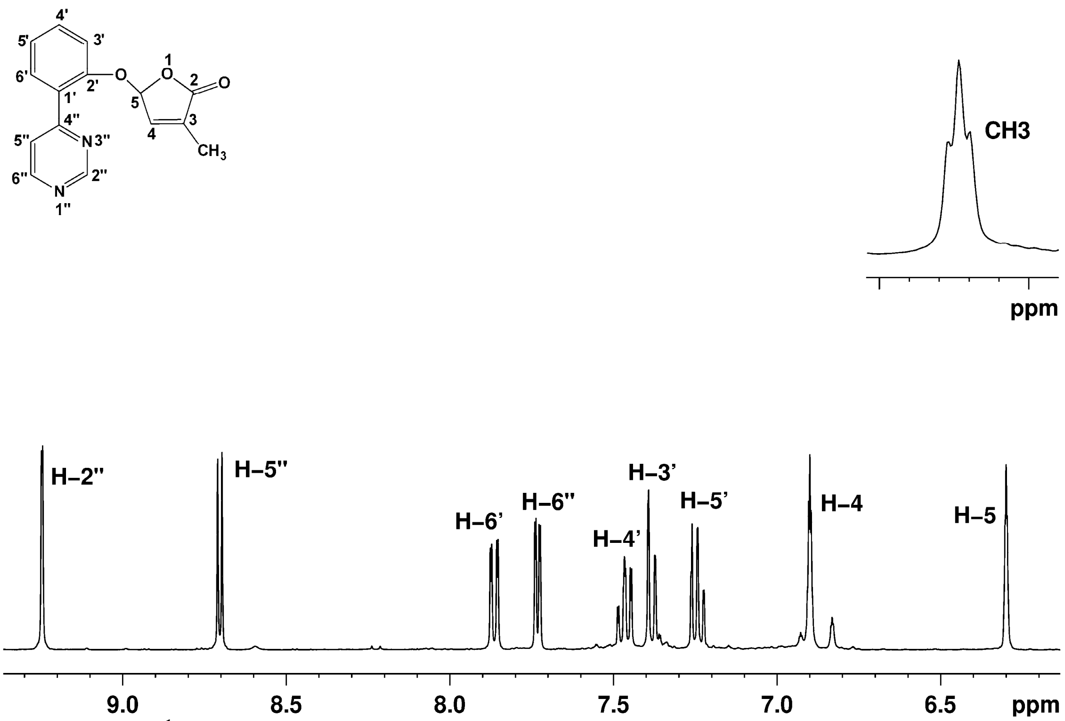

Figure S1. The ^{1}H NMR spectrum for compound **3**, recorded in $CDCl_3$, showing the assignments of the protons. The signal corresponding to the methyl group is displayed in the insert.

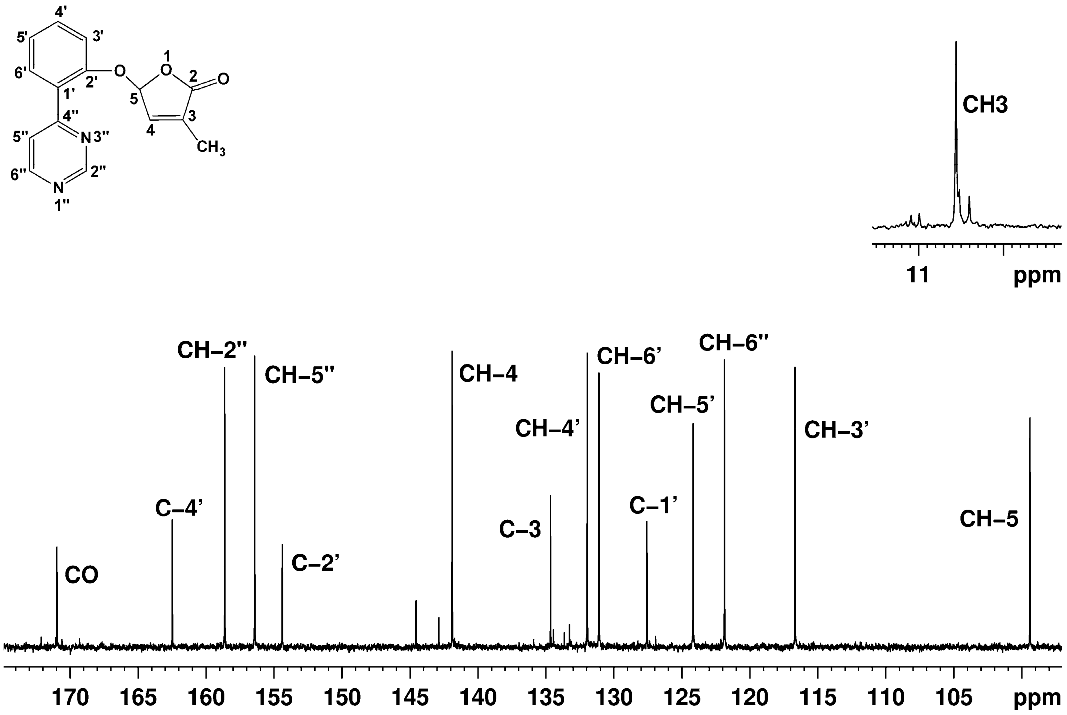

Figure S2. The ^{13}C NMR spectrum for compound **3**, recorded in $CDCl_3$, showing the assignments of the carbons. The signal corresponding to the methyl group is displayed in the insert.

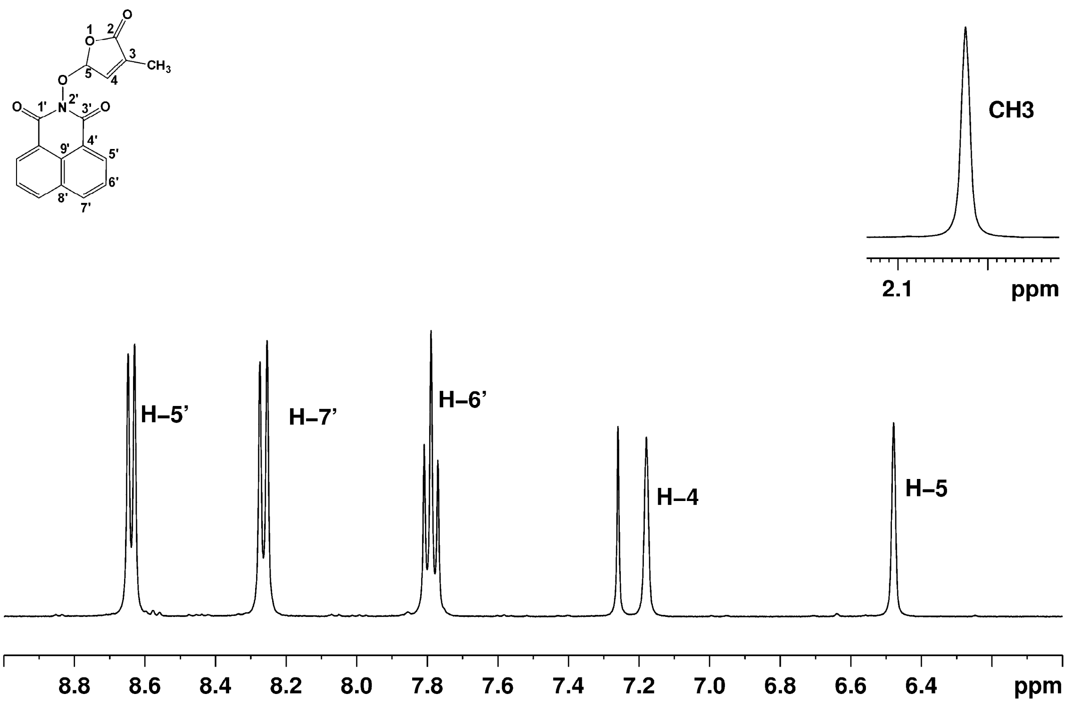

Figure S3. The ^{1}H NMR spectrum for compound **5**, recorded in $CDCl_3$, showing the assignments of the protons. The signal corresponding to the methyl group is displayed in the insert.

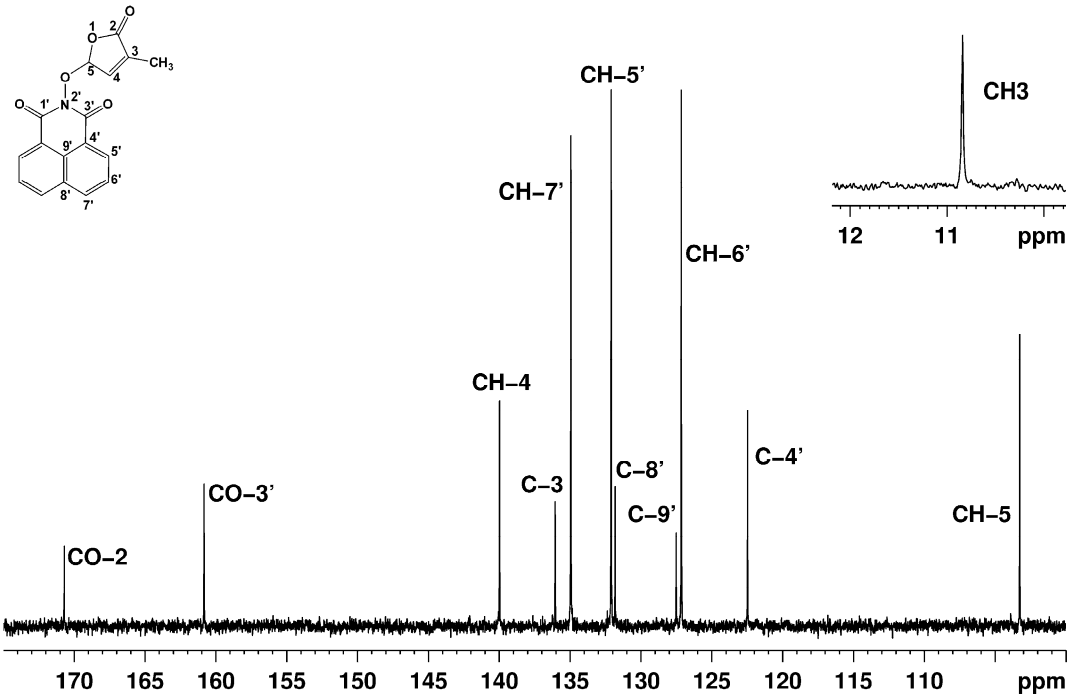

Figure S4. The ^{13}C NMR spectrum for compound **5**, recorded in $CDCl_3$, showing the assignments of the carbons. The signal corresponding to the methyl group is displayed in the insert.

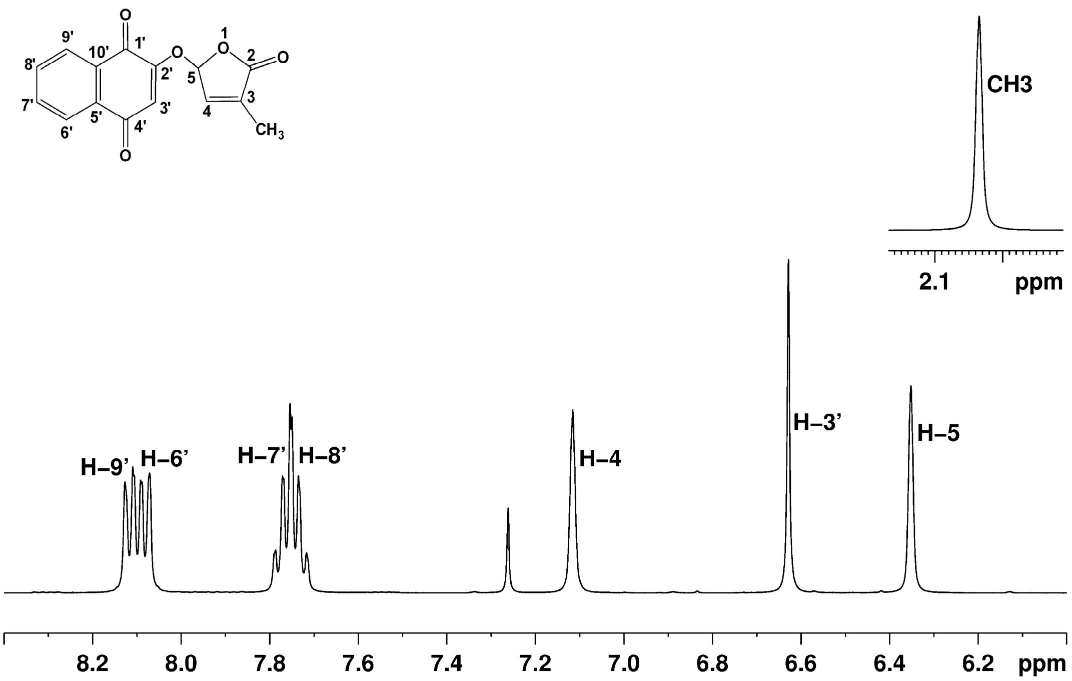

Figure S5. The ^{1}H NMR spectrum for compound **7**, recorded in $CDCl_3$, showing the assignments of the protons. The signal corresponding to the methyl group is displayed in the insert.

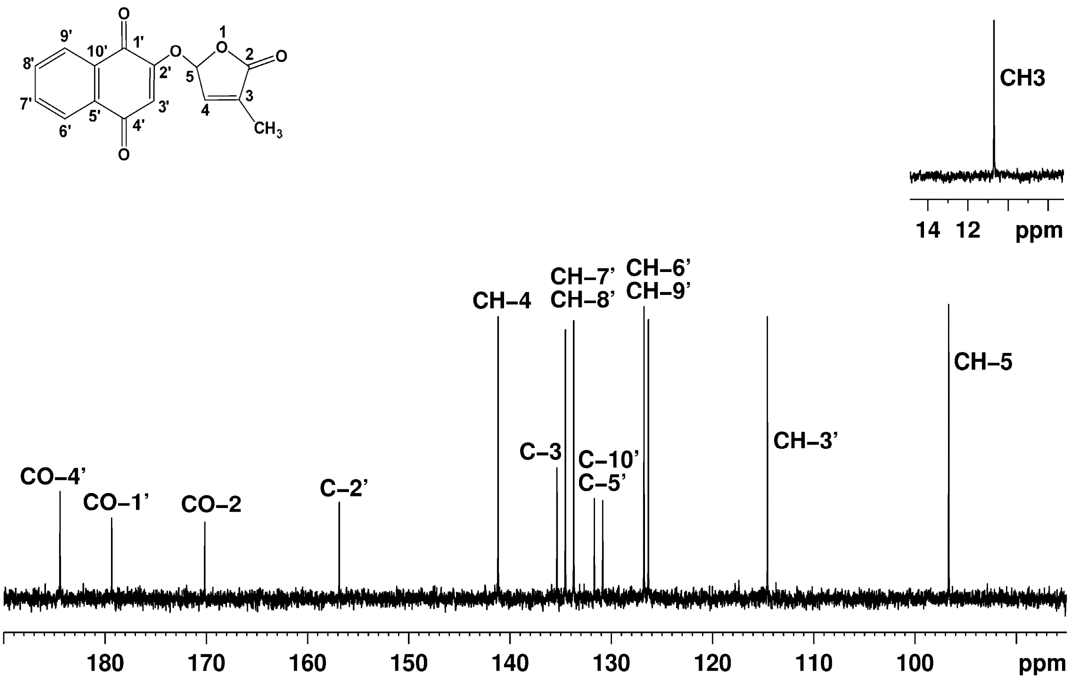

Figure S6. The ^{13}C NMR spectrum for compound **7**, recorded in $CDCl_3$, showing the assignments of the carbons. The signal corresponding to the methyl group is displayed in the insert.
